# Complete resection of a circumferential distal duodenum lesion by endoscopic submucosal dissection

**DOI:** 10.1055/a-2155-3179

**Published:** 2023-09-15

**Authors:** Masami Omae, Francisco Baldaque-Silva, Naining Wang, Toshio Uraoka

**Affiliations:** 1 Division of Medicine, Department of Upper Gastrointestinal Diseases, Karolinska University Hospital and Karolinska Institute, Stockholm, Sweden; 2 Advanced Endoscopy Center Carlos Moreira da Silva, Department of Gastroenterology, Pedro Hispano Hospital, Matosinhos, Portugal; 3 Department of Pathology, Karolinska University Hospital and Karolinska Institute, Stockholm, Sweden; 4 Department of Gastroenterology and Hepatology, Gunma University Graduate School of Medicine, Gunma, Japan

A 71-year-old woman with a lesion extending around the entire luminal circumference along a 10-cm length of the distal duodenum was referred to us at Karolinska University Hospital. Esophagogastroduodenoscopy (EGD) revealed an irregular, heterogeneous, flat lesion ( Fig. 1 , Fig. 2 ). The surface structure had an irregular microsurface pattern but without irregular microvascular pattern. These findings were compatible with superficial duodenal adenoma with low grade dysplasia (LGD). Biopsy specimen confirmed adenoma with LGD. Multidisciplinary conference recommended endoscopic submucosal dissection (ESD).

**Fig. 1:**
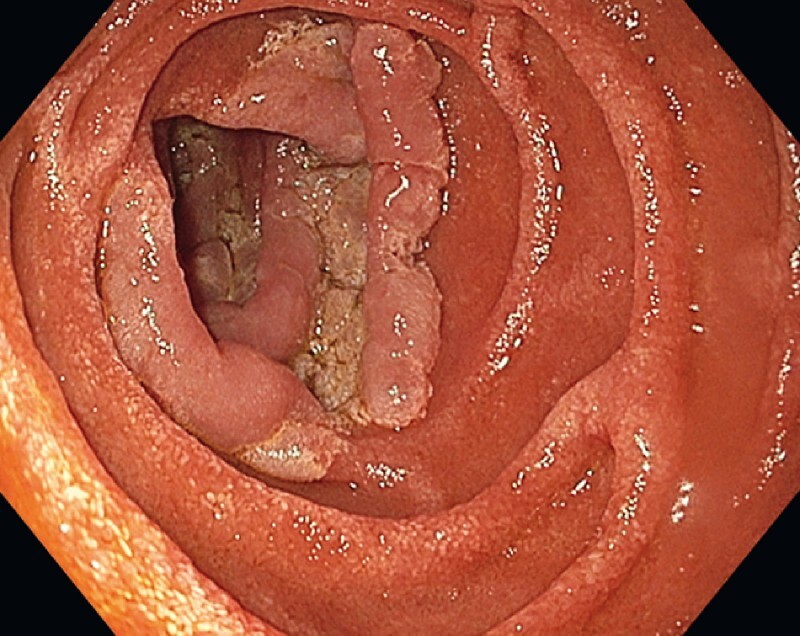
White-light imaging showing an irregular, reddish, and whitish flat elevated mucosa along a 10-cm length of the distal duodenum. The proximal side of the lesion.

**Fig. 2:**
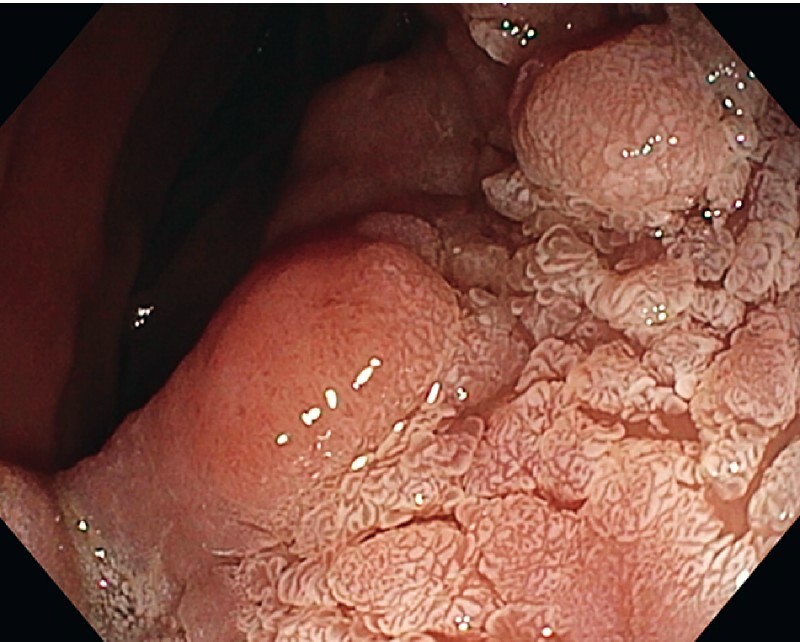
The distal side of the lesion.

A colonoscope (PCF-H190TI; Olympus, Hamburg, Germany) was used with an ST Hood (Fujifilm, Tokyo, Japan). Mucosal incision was performed, starting distally and progressing to the proximal side. The tunnel technique was used, and three tunnels were created. Complete ESD was performed and the lesion was resected en bloc ( Fig. 3 ). The circumferential mucosal defect after ESD was covered with PuraStat (3 D Matrix, London, UK) to prevent delayed bleeding ( Fig. 4 , Video 1 ).

**Fig. 3:**
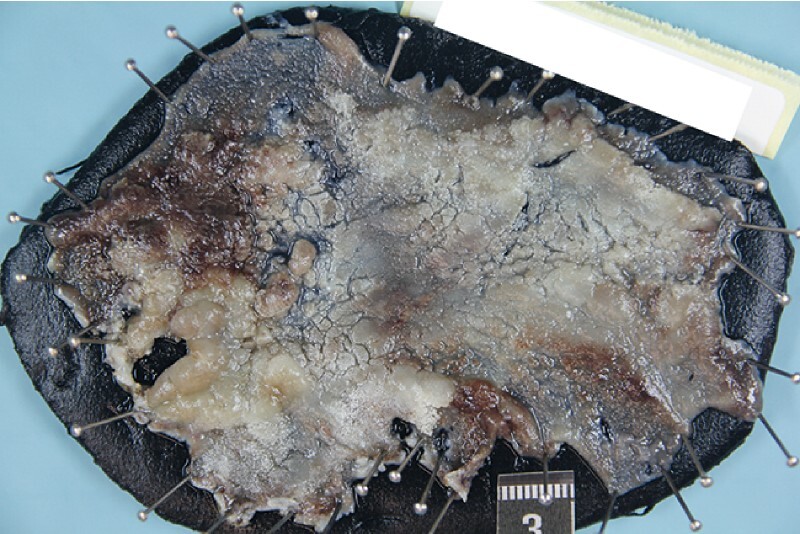
The resected specimen, 100 × 70 mm, fixed in formalin.

**Fig. 4:**
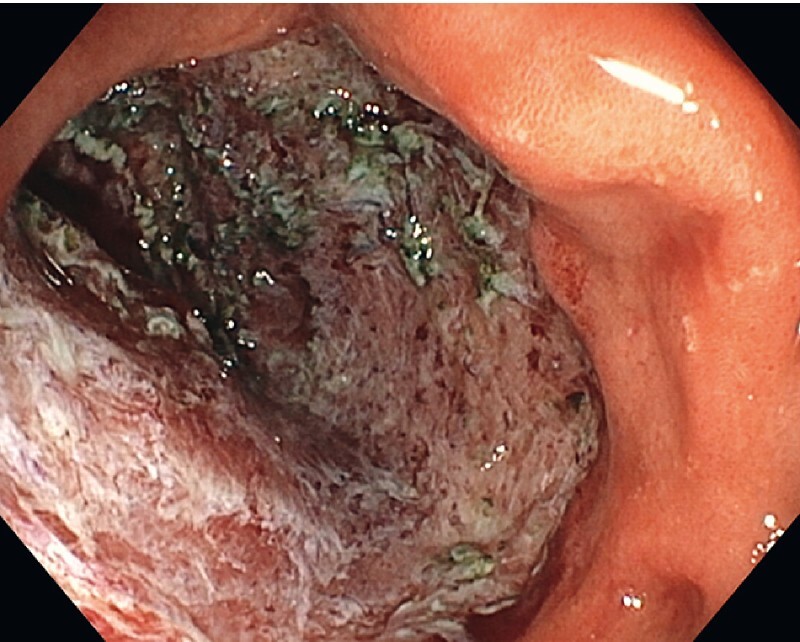
The mucosal defect after endoscopic submucosal dissection.

**Video 1** Endoscopic submucosal dissection of a large circumferential adenoma in the descending duodenum.


The patient started fluid intake on Day 1 and was discharged on Day 3. Oral prednisolone was started on Day 1, for a total of 6 weeks, to prevent stricture formation. The pathological analysis of the resected specimen showed an adenoma with LGD and negative horizontal and vertical margins ( Fig. 5 ). EGD 4 months later showed the ESD scar without any signs of stricture.

**Fig. 5:**
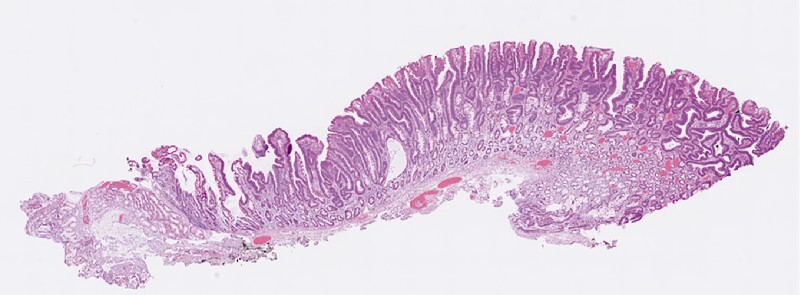
The pathological analysis of the resected specimen showed an adenoma with low grade dysplasia and negative horizontal and vertical margins.

Duodenal lesions involving the entire luminal circumference are rare. To our best knowledge, there are only two reports describing piecemeal endoscopic mucosal resection and laparoscopic and endoscopic cooperative surgery for circumferential superficial nonampullary duodenal epithelial tumors (SNADET) 1 2 . Our case is the first report of a circumferential ESD for the treatment of SNADET, and demonstrates that this technique might be an option in the appropriate setting. Duodenal ESD requires extremely high endoscopic skills and is challenging even for ESD experts 3 . This case shows that ESD might be an option for large duodenal lesions in expert centers and by expert endoscopists in the field.

Endoscopy_UCTN_Code_TTT_1AO_2AG

## Notes

Endoscopy E-Videos

https://eref.thieme.de/e-videos
*E-Videos* is an open access online section of the journal *Endoscopy* , reporting on interesting cases and new techniques in gastroenterological endoscopy. All papers include a high-quality video and are published with a Creative Commons CC-BY license. Endoscopy E-Videos qualify for HINARI discounts and waivers and eligibility is automatically checked during the submission process. We grant 100% waivers to articles whose corresponding authors are based in Group A countries and 50% waivers to those who are based in Group B countries as classified by Research4Life (see: https://www.research4life.org/access/eligibility/ ). This section has its own submission website at https://mc.manuscriptcentral.com/e-videos
